# Insulin-Like Growth Factor-1: A Promising Therapeutic Target for Peripheral Nerve Injury

**DOI:** 10.3389/fbioe.2021.695850

**Published:** 2021-06-24

**Authors:** Benjamin R. Slavin, Karim A. Sarhane, Nicholas von Guionneau, Phillip J. Hanwright, Chenhu Qiu, Hai-Quan Mao, Ahmet Höke, Sami H. Tuffaha

**Affiliations:** ^1^Department of Plastic and Reconstructive Surgery, School of Medicine, Johns Hopkins University, Baltimore, MD, United States; ^2^Division of Plastic and Reconstructive Surgery, University of Miami Miller School of Medicine, Miami, FL, United States; ^3^Department of Materials Science and Engineering, Whiting School of Engineering, Johns Hopkins University, Baltimore, MD, United States; ^4^Institute for Nanobiotechnology, Johns Hopkins University, Baltimore, MD, United States; ^5^Department of Biomedical Engineering, School of Medicine, Johns Hopkins University, Baltimore, MD, United States; ^6^Translational Tissue Engineering Center, School of Medicine, Johns Hopkins University, Baltimore, MD, United States; ^7^Department of Neurology, School of Medicine, Johns Hopkins University, Baltimore, MD, United States; ^8^Department of Neuroscience, School of Medicine, Johns Hopkins University, Baltimore, MD, United States

**Keywords:** IGF-1, PNI, peripheral nerve injury, nerve regeneration, somatomedin C, nanoparticle carrier

## Abstract

Patients who sustain peripheral nerve injuries (PNIs) are often left with debilitating sensory and motor loss. Presently, there is a lack of clinically available therapeutics that can be given as an adjunct to surgical repair to enhance the regenerative process. Insulin-like growth factor-1 (IGF-1) represents a promising therapeutic target to meet this need, given its well-described trophic and anti-apoptotic effects on neurons, Schwann cells (SCs), and myocytes. Here, we review the literature regarding the therapeutic potential of IGF-1 in PNI. We appraised the literature for the various approaches of IGF-1 administration with the aim of identifying which are the most promising in offering a pathway toward clinical application. We also sought to determine the optimal reported dosage ranges for the various delivery approaches that have been investigated.

## Introduction

Peripheral nerve injuries (PNIs) affect approximately 67 800 people annually in the United States alone (Wujek and Lasek, 1983; Noble et al., 1998; Taylor et al., 2008). Despite optimal management, many patients experience lasting motor and sensory deficits, the majority of whom are unable to return to work within 1 year of the injury (Wujek and Lasek, 1983). The lack of clinically available therapeutic options to enhance nerve regeneration and functional recovery remains a major challenge.

The amount of time that elapses between initial nerve injury and end-organ reinnervation has consistently been shown to be the most important predictor of functional recovery following PNI (Scheib and Hoke, 2013), with proximal injuries and delayed repairs resulting in worse outcomes (Carlson et al., 1996; Tuffaha et al., 2016b). This is primarily due to denervation-induced atrophy of muscle and Schwann cells (SCs) (Fu and Gordon, 1995). Following surgical repair, axons often must regenerate over long distances at a relatively slow rate of 1–3 mm/day to reach and reinnervate distal motor endplates. Throughout this process, denervated muscle undergoes irreversible loss of myofibrils and loss of neuromuscular junctions (NMJs), thereby resulting in progressive and permanent muscle atrophy. It is well known that the degree of muscle atrophy increases with the duration of denervation (Ishii et al., 1994). Chronically denervated SCs within the distal nerve are also subject to time-dependent senescence. Following injury, proliferating SCs initially maintain the basal lamina tubes through which regenerating axons travel. SCs also secrete numerous neurotrophic factors that stimulate and guide axonal regeneration. However, as time elapses without axonal interaction, SCs gradually lose the capacity to perform these important functions, and the distal regenerative pathway becomes inhospitable to recovering axons (Ishii et al., 1993; Glazner and Ishii, 1995; Grinsell and Keating, 2014).

Research efforts to improve PNI outcomes have primarily focused on isolated processes, including the acceleration of intrinsic axonal outgrowth and maintenance of the distal regenerative environment. In order to maximize functional recovery, a multifaceted therapeutic approach that both limits the damaging effects of denervation atrophy on muscle and SCs and accelerates axonal regeneration is needed.

A number of promising potential therapies have been under investigation for PNI. Many such experimental therapies are growth factors including glial cell line-derived neurotrophic factor (GDNF), fibroblast growth factor (FGF), and brain-derived neurotrophic growth factor (Fex Svenningsen and Kanje, 1996; Lee et al., 2007; Gordon, 2009). Tacrolimus (FK506), delivered either systemically or locally, has also shown promise in a number of studies (Konofaos and Terzis, 2013; Davis et al., 2019; Tajdaran et al., 2019).

Insulin-like growth factor-1 (IGF-1) is a particularly promising candidate for clinical translation because it has the potential to address the need for improved nerve regeneration while simultaneously acting on denervated muscle to limit denervation-induced atrophy. However, like other growth factors, IGF-1 has a short half-life of 5 min, relatively low molecular weight (7.6 kDa), and high water-solubility: all of which present significant obstacles to therapeutic delivery in a clinically practical fashion (Gold et al., 1995; Lee et al., 2003; Wood et al., 2009). Here, we present a comprehensive review of the literature describing the trophic effects of IGF-1 on neurons, myocytes, and SCs. We then critically evaluate the various therapeutic modalities used to upregulate endogenous IGF-1 or deliver exogenous IGF-1 in translational models of PNI, with a special emphasis on emerging bioengineered drug delivery systems. Lastly, we analyze the optimal dosage ranges identified for each mechanism of IGF-1 with the goal of further elucidating a model for future clinical translation.

## Methods

We comprehensively reviewed the literature for original studies examining the efficacy of IGF-1 in treating PNI. We queried the PubMed and Embase databases for terms including “Insulin-Like Growth Factor I,” “IGF1,” “IGF-1,” “somatomedin C,” “PNIs,” “peripheral nerves,” “nerve injury,” “nerve damage,” “nerve trauma,” “nerve crush,” “nerve regeneration,” and “nerve repair.” Following title review, our search yielded 218 results. Inclusion criteria included original basic science studies utilizing IGF-1 as a means of addressing PNI. Following abstract review, 56 studies were sorted by study type and mechanism of delivery into the following categories: (1) *in vitro*, (2) *in vivo* endogenous upregulation of IGF-1, or (3) *in vivo* delivery of exogenous IGF-1. Studies included in the *in vivo* exogenous IGF-1 group were further sub-stratified into systemic or local delivery, and the local IGF-1 delivery methods were further sub-divided into free IGF-1 injection, hydrogel, or mini-pump studies. Following categorization by mechanism of IGF-1 delivery, the optimal dosage range for each group was calculated by converting all reported IGF-1 dosages to nM for ease of comparison using the standard molecular weight of IGF-1 of 7649 Daltons. After standardization of dosages to nM, the IGF-1 concentration reported as optimal from each study was used to calculate the overall mean, median, and range of optimal IGF-1 dosage for each group.

## *In Vitro* Effects of IGF-1 on Neurons, Schwann Cells, and Myocytes

The positive trophic and anti-apoptotic effects of IGF-1 are primarily mediated via the PI3K-Akt and MAP-kinase pathways (Ho and 2007 GH Deficiency Consensus Workshop Participants, 2007; Chang et al., 2017). Autophosphorylation of the intracellular domain of IGF-1 receptors results in the activation of insulin receptor substrates 1–4, followed by activation of Ras GTPase, and then the successive triggering of Raf, MEK, and lastly ERK. Through activation of Bcl-2, ERK has been shown to prevent apoptosis and foster neurite growth. Ras activation also triggers aPKC and Akt (Homs et al., 2014), with the active form of the latter inhibiting GSK-3β and thus inhibiting a number of pro-apoptotic pathways (Kanje et al., 1988; Schumacher et al., 1993; Chang et al., 2017). Additionally, the JAK-STAT pathway is an important contributor toward the stimulation of neuronal outgrowth and survival by facilitating Growth Hormone (GH) receptor binding on target tissue to induce IGF-1 release (Meghani et al., 1993; Cheng et al., 1996; Seki et al., 2010; Chang et al., 2017). These biochemical mechanisms enable GH and IGF-1 to exert anabolic and anti-apoptotic effects on neurons, SCs, and myocytes (Tuffaha et al., 2016b).

A number of *in vitro* studies have highlighted the neurotrophic effects of IGF-1 (Table 1). Using cultured nerve, SCs, and dorsal root ganglion (DRG) cells, these studies demonstrate that IGF-1 promotes neurite outgrowth and limits neuronal apoptosis (Caroni and Grandes, 1990; Sumantran and Feldman, 1993; Akahori and Horie, 1997; Delaney et al., 2001; Ogata et al., 2004; Liang et al., 2007; Scheib and Hoke, 2013, 2016a,b). Additionally, several *in vitro* studies have shown that IGF-1 supports SC myelination and inhibits SC apoptosis whilst also stimulating nerve sprouting into denervated muscle and reducing muscle atrophy (Caroni and Grandes, 1990; Sumantran and Feldman, 1993; Ogata et al., 2004; Liang et al., 2007; Scheib and Hoke, 2016a,b).

**TABLE 1:** *In vitro* studies of IGF-1 on neurons, SCs, and DRG cells (IGF-1, insulin-like growth factor 1; SC, schwann cells; DRG, dorsal root ganglion, bFGF, basic fibroblast growth factor; Bcl-xL, B-cell lymphoma-extra large; C-myc, cellular-myc; GAP-43, growth-associated protein 43; GGF, glial growth factor).

**Cell Target**	**IGF-1 Concentrations Tested (in originally concentrations)**	**Optimal IGF-1 Concentration Reported (standardized to nM)**	**Key Finding (Citation)**
Neurons	10 and 100 ng/mL	1.31	Insulin, IGF-1 and 2 are mitogenic to cultured rat sciatic nerve segments, stimulate [3H]thymidine incorporation through their receptors Ho and 2007 GH Deficiency Consensus Workshop Participants, 2007.
Neurons/Myocytes	0.1, 0.3, 0.5, 2, and 10 nM	2	Nerve sprouting in innervated adult skeletal muscle is induced by exposure to elevated levels of insulin-like growth factors Gorio et al., 2001.
SCs	20 ng/mL	2.61	IGF-1 serves as a mitogen for rat SCs Wood et al., 2009.
SCs	0.1, 0.3, 1.0, and 3.0 nM	3	IGF-1 induces mitogenesis in cultured SCs Gold et al., 1995.
DRGs	(50 or 100 ng/mL IGF-1) + (5, 10, 20, or 50 ng/mL bFGF)	6.54	FGF and insulin-like growth factor rescue growth cones of sensory neurites from collapse after tetracaine-induced injury Chang et al., 2017.
DRGs	1–10 nM IGF-1	10	IGF-I enhances neurite regeneration but is not required for its survival in adult DRG explant Schumacher et al., 1993.
SCs	0.3 and 10 nM	10	Insulin-like growth factor-I and over-expression of Bcl-xL prevent glucose-mediated apoptosis in Schwann cells Kanje et al., 1988.
Neurons	0, 3, and 10 nM	10	Insulin-like growth factor I regulates c-myc and GAP-43 messenger ribonucleic acid expression in SH-SY5Y human neuroblastoma cells Delaney et al., 2001.
Neurons	0.1, 1.0, 3.0, and 10 nM	10	Insulin-like growth factor has a positive trophic effect on human neuroblastoma cell growth Homs et al., 2014.
SCs	10, (100 ng/mL IGF-1 + 20 ng/mL GGF)	13.07	Extracellular control of cell size can be achieved through combination of IGF + GGF to augment cell cycle progression Scheib and Hoke, 2013.
SCs	150 ng/mL	19.61	IGF1 has a positive trophic effect on SC myelination Cheng et al., 1996.
SCs	20 nM	20	IGF1 stimulates *de novo* fatty acid synthesis for SC myelination Seki et al., 2010.

The neurotrophic effects of IGF-1 have been found to be dose-dependent and independent of cell-cycle stage (Sumantran and Feldman, 1993; Tuffaha et al., 2016b). Specific trophic benefits to neurons include the promotion of neurite outgrowth, prevention of neuronal apoptosis, and the promotion of growth cone motility. As the proximal end of an injured nerve begins to recover, regenerating axons are guided to reinnervate their distal targets by numerous chemotrophic factors, resulting in the formation of a growth cone. IGF-1 plays a key role in the motility of the growth cone by inducing reorganization of actin and activation of focal adhesion molecules via the PI3K/Akt pathway (Tuffaha et al., 2016b). IGF-1 further augments growth cone motility via downregulation of c-myc, a cell proliferation transcription factor indicative of neuronal differentiation, and upregulation of growth cone-associated protein 43 (GAP-43), a vital component of neurite formation.

Schwann cells are instrumental to recovery following PNI given their ability to support and guide axonal regeneration via the secretion of neurotrophic factors and maintenance of basal lamina tubes (Scheib and Hoke, 2013, 2016a,b; Tuffaha et al., 2016b). Initially after injury, myelinating SCs distal to the site of injury undergo conversion to a more immature, proliferating repair phenotype (Nocera and Jacob, 2020). Throughout this process, SCs express a variety of genes that dynamically control the regenerative process by promoting survival of neurons, breakdown of damaged axons, clearance of myelin, axonal regrowth, and guidance to the axons’ former targets, finally leading to remyelination of the regenerated axon (Chen et al., 2015; Gordon, 2020; Nocera and Jacob, 2020). Unfortunately, upregulation of pro-regenerative gene expression is temporary and the SCs gradually lose the continued ability to support axonal regrowth as time elapses without axonal interaction (Gordon, 2020). A more detailed description of the biological processes underpinning the role of SCs in peripheral nerve regeneration can be found in a recent review article by Nocera and Jacob (2020). IGF-1 supports SCs by promoting their proliferation, maturation, and differentiation to myelinating phenotypes, while concurrently inhibiting SC apoptosis via the PI3K pathway (Scheib and Hoke, 2013; Tuffaha et al., 2016b). IGF-1’s ability to initiate myelination centers around regulating the balance between ERK, a pathway suppressing SC differentiation, and PI3K-Akt, a pathway promoting SC differentiation via increased expression of myelin basic protein and myelin-associated glycoprotein (Schumacher et al., 1993; Stewart et al., 1996; Conlon et al., 2001; Scheib and Hoke, 2016a).

Peripheral nerve injury subjects muscle to prolonged denervation that results in myofiber atrophy with increased proteolysis, decreased contractility, and interstitial fibrosis. As the period of denervation extends, these proteolytic and fibrotic processes continue, thereby decreasing the viability of muscle to accept regenerating axons (Shavlakadze et al., 2005; Tuffaha et al., 2016b). In addition to the deleterious effects of prolonged denervation and fibrosis on muscle, functional recovery is hindered by the failure of regenerating motor nerve fibers to come into contact with the specific motor pathways that guide them back to their original motor endplates (Gordon, 2020). A more thorough description of the biological processes and pathways implicated in denervation-induced muscle atrophy can be found in this recent review article by Ehmsen and Hoke (2020). Following nerve injury, local levels of IGF-1 increase and stimulate axonal sprouting into denervated muscle (Homs et al., 2014). IGF-1 also activates the Akt/mTOR pathway, thereby decreasing atrophy markers including MAFbx and MuRF1 (Bodine et al., 2001; Stitt et al., 2004). Also of note, IGF-1’s propensity for decreasing inflammation via promotion of a pro-regenerative M2 macrophage shift over pro-inflammatory M1 reduces the degree of scarring and fibrosis that could otherwise interfere with the targeting of regenerating motor axons (Labandeira-Garcia et al., 2017; Zhao et al., 2021).

Studies included in Table 1 tested an array of dosages of *in vitro* IGF-1. After considering the individual results of each study, we found that many reported a maximally efficacious IGF-1 dosage. Using each of the reported dosage ranges with the highest efficacy from the *in vitro* studies, we estimated the optimal concentration range for *in vitro* IGF-1 to be 2.61–20.0 nM (mean = 9.81 nM, median = 10.0 nM) (Rinderknecht and Humbel, 1978).

## *In Vivo* Upregulation of Endogenous IGF-1

Many of the *in vitro* benefits of IGF-1 to neurons, SCs, and myocytes have also been observed *in vivo*. IGF-1 is produced endogenously by the liver. There has also been documentation of autocrine and paracrine IGF-1 production by multiple cell and tissue types including SCs and myocytes (Laron, 2001; McMullen et al., 2004; Apel et al., 2010). Multiple studies have found that following PNI, IGF-1 increases axon number and maintains SC proliferation at near-normal levels while also enhancing NMJ recovery to promote end-organ reinnervation (Caroni and Grandes, 1990; Kanje et al., 1991; Apel et al., 2010; Emel et al., 2011; Bayrak et al., 2017). Studies administering anti-IGF-1 antibodies to a sciatic nerve crush model further validated the role of IGF-1 in PNI, finding a diminished capacity for regeneration (Kanje et al., 1989; Sjoberg and Kanje, 1989).

One strategy that has been used to take advantage of the therapeutic benefits of IGF-1 involves systemic upregulation of endogenous IGF-1 production at the protein level in injured nerves via upstream augmentation of the GH axis (Table 2; Kanje et al., 1988; Gorio et al., 1998, 2001; Losa et al., 1999; Madaschi et al., 2003; Flint et al., 2004; Rabinovsky and Draghia-Akli, 2004; Saceda et al., 2011; Bagriyanik et al., 2014; Homs et al., 2014; Nagata et al., 2014; Wang et al., 2015; Tsai et al., 2016; Tuffaha et al., 2016a; Chang et al., 2017; Lopez et al., 2019). The most straightforward approach to accomplish this aim involves systemic administration of GH (Saceda et al., 2011; Tuffaha et al., 2016a; Lopez et al., 2019). This has been shown to be efficacious in both acute PNI repair models and also in a model in which chronic denervation is induced prior to nerve repair (Lopez et al., 2019). One of the strengths of this approach is that it offers a clear pathway to clinical translation, as GH is already available as an FDA-approved drug for other indications. However, it is limited by the need for systemic treatment, which requires daily parenteral dosing and carries a number of side effects, in addition to a lack of fine control over local IGF-1 levels.

**TABLE 2:** *In vivo* studies using upregulators of endogenous IGF-1 (AAVrh, adeno-associated virus rhesus isolate; EPO, erythropoietin; GAGs, glycosaminoglycans; pDNA, plasmid DNA; hIGF-1, human IGF-1; GH, growth hormone; HGH, human growth hormone).

**Cell Target**	**Local Delivery?**	**Endogenous IGF-1 Upregulator Dosage and Delivery**	**Specific GH Axis Modifier (Citation)**
Nerve	Y/N	400 nL/min AAVrh10 via “micropump” directly into sciatic nerve and also intrathecally	AAVrh10 Virus delivery of IGF-1 Tajdaran et al., 2019
Nerve	Y	30, 60, 100, 150, and 200 mg/mL/kg Alpinia oxyphylla fruit locally applied to nerve gap	Alpinia oxyphylla Bodine et al., 2001
Nerve	Y	5000 U/kg EPO	EPO Apel et al., 2010
Nerve/Muscle	N	1 mg/kg GAGs (64.4% heparin, 28.8% dermatan sulfate, and 6.7% chondroitin sulfate) injected intraperitoneally daily	GAGs Kanje et al., 1991
Nerve/Muscle	N	5 mg/kg GAGs via daily intraperitoneal injections (64.4% heparin, 28.8% dermatan sulfate, and 6.7% chondroitin sulfate)	GAGs Bayrak et al., 2017
Nerve/Muscle	N	1 mg/kg GAGs (64.4% heparin, 28.8% dermatan sulfate, and 6.7% chondroitin sulfate)	GAGs Sjoberg and Kanje, 1989
Nerve/Muscle	Y	134 ug/mL IGF-1-expressing pDNA, delivered via biocompatible polyplex nanomicelle	Gene Delivery Labandeira-Garcia et al., 2017
Nerve/Muscle	N	0.35 mg/kg myostatin propeptide plasmid injected at five different locations on shaved rat abdomen	Gene Delivery Zhao et al., 2021
Nerve/Muscle	Y	30 uL hIGF-1 vector solution (90 ug DNA)	Gene Transfer Laron, 2001
Nerve	N	0.4 mg/day subcutaneous GH injection	GH Chang et al., 2017
Nerve	N	0.1 mg/kg/day subcutaneous GH injection	GH Kanje et al., 1989
Nerve	N	0.6 mg/day subcutaneous GH injection	GH Homs et al., 2014
Nerve	N	400 mIU/day HGH via mini pump	GH Lee et al., 2003
Nerve/Muscle	N	1 mg/kg intraperitoneal heparin injections daily	Heparin Emel et al., 2011
Muscle	Y	Plasmid-IGF-1 delivery via intramuscular injection increased blood flow and angiogenesis in diabetic/diseased limb	Plasmid Therapy Rinderknecht and Humbel, 1978
Nerve	N	10 mg/kg injections of resveratrol for 14 days	Resveratrol McMullen et al., 2004

Heparin is another upregulator of endogenous IGF-1 that was shown to be effective in promoting nerve and muscle recovery following PNI, as demonstrated by Madaschi et al. (2003) with intraperitoneal injection of a dosage of 1 mg/kg (Madaschi et al., 2003). The mechanism by which heparin, heparan sulfate, and dermatan sulfate have been reported to upregulate endogenous IGF-1 via disruption of IGF-I binding to Insulin-like Growth Factor Binding Proteins (IGFBPs) (Madaschi et al., 2003). Heparin is also thought to inhibit the binding of IGFBP-3 to extracellular matrix heparan sulfate proteoglycans, thereby reducing the affinity of IGFBPs for IGF-I administration and resulting in the release of IGFBP-3 from the cell surface (Gorio et al., 2001). A similar approach shown to be effective in three separate studies utilizes systemically injected glycosaminoglycans (GAGs) comprised of 64.4% heparin, 28.8% dermatan sulfate, and 6.7% chondroitin sulfate. The effectiveness of GAGs in enhancing the recovery process following PNI was evidenced by a marked increase in IGF-1 levels in denervated muscle, leading to enhanced recovery as measured by nerve-evoked muscle force testing and the extent of muscle reinnervation (Gorio et al., 1998, 2001; Losa et al., 1999).

Gene delivery targeted to skeletal myocytes has also demonstrated promise as a method of upregulating IGF-1 production in PNI models (Flint et al., 2004; Rabinovsky and Draghia-Akli, 2004; Nagata et al., 2014; Tsai et al., 2016). This approach has been applied both systemically as well as directly to the local site of PNI. Amongst the gene delivery protocols included in Table 2, the work of Nagata et al. (2014) is notable given its use of a biocompatible polyplex nanomicelle as a means of delivering IGF-1 plasmid DNA (pDNA) to the local site of PNI (Nagata et al., 2014). The diverse strategies employed by these systemic GH axis modifiers demonstrate the flexibility with which IGF-1 can potentially be incorporated into future translational approaches. However, these systemic therapeutic approaches are all limited by the resulting systemic upregulation of IGF-1 with the associated risks and side effects as well as the lack of fine control of IGF-1 levels within the target tissues, specifically the injured nerve and denervated muscle.

## Delivery of Exogenous IGF-1

Both systemic and local techniques have been tested for exogenous IGF-1 administration. Local delivery was further categorized into three different approaches: targeted injection of free IGF-1, implantable mini-pumps, and IGF-1-eluting hydrogels. As with the *in vitro* studies, reported optimal dosages varied. When possible, we calculated an optimal concentration mean, median, and range for each method of *in vivo* IGF-1 administration.

### Systemic *in vivo* Delivery of IGF-1

Systemic delivery of IGF-1 is achieved via either daily subcutaneous or intraperitoneal injections of free IGF-1. Reported optimal dosages for regeneration of nerve, SC, and muscle range from 0.001 to 1.00 mg/kg/day with a mean of 0.59 mg/kg/day and a median of 0.75 mg/kg/day of IGF-1 (Contreras et al., 1993, 1995; Vaught et al., 1996; Vergani et al., 1998; Lutz et al., 1999; Mohammadi and Saadati, 2014; Table 3). The calculated mean and median IGF-1 concentrations for systemic delivery were the highest of any of the delivery mechanisms included in our analysis. This finding emphasizes that the use of a systemic approach necessitates greater dosages of IGF-1 to account for off-target distribution and degradation/clearance prior to reaching the injury site. Notably, almost none of the systemic studies included in this analysis quantified the concentration of IGF-1 at the target injury site, which raises significant concerns about the validity of the findings. With regards to clinical applicability, systemic IGF-1 delivery is severely limited by the risk of side effects, including hypoglycemia, lymphoid hyperplasia, body fat accumulation, electrolyte imbalances, and mental status changes (Elijah et al., 2011; Tuffaha et al., 2016b; Vilar et al., 2017). In contrast to upregulation of systemic IGF-1 via GH Releasing Hormone (GHRH), treatment with systemic IGF-1 does not have the benefit of upstream negative feedback control and therefore poses a greater risk of resulting in spiking IGF-1 levels.

**TABLE 3:** *In vivo* studies using systemic administration of IGF-1 (rhIGF-1, recombinant human IGF-1; subq, subcutaneously).

**Cell Target**	**IGF-1 Dosage**	**Optimal Reported Dosage of IGF-1 Injected Daily (in mg/kg/day) (Citation)**
Nerve/Muscle	100 ng/kg/day intraperitoneal injections	0.0001 Nagata et al., 2014
Nerve/Muscle	0.02 mg/kg/day, 1 mg/kg/day	0.02 Tsai et al., 2016
Nerve	0.5 mg/kg rhIGF-1	0.50 Rabinovsky and Draghia-Akli, 2004
Nerve	0.1, 0.3, 1, 3, 10, and 30 mg/kg/day rhIGF-1 for 14 days	1.00 Flint et al., 2004
Nerve	1 mg/kg subq for delay of grip strength deterioration, 0.3–1.0 mg/kg subq for motor/sensory neuroprotection	1.00 Wang et al., 2015
Nerve	1 mg/kg subq	1.00 Bagriyanik et al., 2014
		

### Local Injection of Free IGF-1

Local administration of IGF-1 was achieved by several targeted approaches including direct application of free IGF-1 to the injured nerve at the time of surgical transection as well as single, periodic, and daily local injections of free IGF-1 to the injury site (Caroni and Grandes, 1990; Welch et al., 1997; Day et al., 2001, 2002; Stitt et al., 2004; Emel et al., 2011; García Medrano et al., 2013; Mohammadi et al., 2013; Gu et al., 2015; Kostereva et al., 2016; Table 4). Local injection of free IGF-1 is not practical for clinical application as the half-life of IGF-1 is 10 min while the time required for regeneration to occur is often many months (Mayocliniclabs.com, 2020). Multiple injections per day would thus be required to maintain local tissue concentrations. We therefore did not attempt to ascertain the optimal dosages for this approach.

**TABLE 4:** *In vivo* studies using targeted local application/injection of IGF-1 (BSA, bovine serum albumin; PBS, phosphate-buffered saline; PRP, platelet-rich plasma; pcDNA, plasmid-cloning DNA; hIGF1, human IGF-1; PNI, peripheral nerve injury; PDGF, platelet-derived growth factor).

**Cell Target**	**IGF-1 Protocol**	**Optimal Quantity IGF-1 Used and Application Regimen**	**Study Results (Citation)**
Nerve	Three sub-epineural 10 uL injections along distal nerve prior to transection, 200 ng/30 mL daily injections to transplanted limb	0.0067 ug/mL daily injection	IGF-1 increased nerve regeneration Losa et al., 1999
Nerve	0.2 nM IGF-1 achieved half-maximal response, 100 ng IGF-1 in 100–200 uL BSA in PBS injected daily for 4, 7, or 14 days	0.1 ug daily injection	IGF-1 induced nerve sprouting Gorio et al., 1998
Nerve	15 ug IGF-1 (0.125 mL) injections/day for 8 days, delivered via injection port at crush-injured nerve site, IGF-1 better than PRP	15 ug daily injection	IGF-1 decreased muscle atrophy Saceda et al., 2011
Nerve/Muscle	Intramuscular injection of 200 ug into the gastrocnemius muscle on the 3rd, 5th, 7th, and 9th day	200 ug periodic injection	IGF-1 partially rescues denervation-induced muscle loss Tuffaha et al., 2016a
Muscle	100 ng at 0, 3, and 7 days post-denervation	0.1 ug periodic injection	IGF-1 resulted in relative preservation of muscle diameter, weight, contractile properties Lopez et al., 2019
Nerve/Muscle	100 ug on day 1, 3, and 7	100 ug periodic injection	IGF-1 decreased muscle atrophy following chronic denervation Mohammadi and Saadati, 2014
Nerve	4 ug/10 uL pcDNA-IGF-1 injected into clamped epineurium immediately	0.4 ug/uL single injection	Exogenous hIGF-1 is capable of protecting spinal cord motoneurons following PNI Lutz et al., 1999
Nerve	Graft filled with 10 uL (30 ng/kg) IGF-1 at time of procedure	0.03 ug/kg single application	IGF-1 accelerated and improved functional recovery and morphometric indices of sciatic nerve Vergani et al., 1998
Nerve	Combination of 1.5 ug IGF-1 and 0.75 ug PDGF	1.5 ug single application paired with 0.75 ug PDGF	Combined PDGF/IGF-1 did not significantly enhance PNI regeneration after 6 weeks Contreras et al., 1995
Nerve	2 mL rhIGF-1 with concentration 10 mg/mL injected into graft site	2.0 × 10^4^ ug single application	47–62% increase in % of nerve endings in distal sciatic region Vaught et al., 1996
			

### Local Mini-Pump Delivery of IGF-1

Mini-osmotic pumps provide a sustained, local delivery of exogenous IGF-1 (Table 5; Kanje et al., 1989; Sjoberg and Kanje, 1989; Ishii and Lupien, 1995; Tiangco et al., 2001; Fansa et al., 2002; Apel et al., 2010; Luo et al., 2016). This technique involves subcutaneous implantation of an osmotic pump in the abdomen with extension of a catheter from the pump to the transected nerve site. The positioning of the catheter is maintained by suturing it to local connective tissue. A fixed concentration and quantity of IGF-1 is then loaded into the pump and released at a constant rate (Kanje et al., 1989).

**TABLE 5:** *In vivo* studies using mini pumps for local administration of IGF-1.

**Cell Target**	**IGF-1 Concentrations**	**Pump Rate (in uL/h)**	**Optimal Concentration of IGF-1 Loaded into Mini-Pump (in ug/mL) (Citation)**
Nerve	100 mg/mL total in the mini pump, continuous pump rate of 0.25 uL/h over 28 days	0.25	1.00 × 10^5^ Elijah et al., 2011
Nerve	100 ug/mL @ 0.25 uL/h (0.025 ug/h) over 12 weeks w/pump replacement at 6 weeks	0.25	100.00 Gordon, 2020
Nerve	50 or 100 ug/mL @ 0.25 uL/h over 28 days (no significant difference between the 50 and 100 ug/mL concentrations initially loaded into mini pump)	0.25	50.00 Vilar et al., 2017
Nerve	0.10 ug/uL @ 0.25 uL/h over 1–4 weeks	0.25	0.10 Kostereva et al., 2016
Nerve	50, 100, 200 ug/mL, 100 or 200 ug/mL “optimal, 25% increase in regeneration” over 6 days	1.05	100.00 Shavlakadze et al., 2005
Nerve	Local mini pump: 10 ug/mL released at a rate of 0.5 uL/h over 7 days Systemic mini pump: 200 ug/mL IGF-1 released at a rate of 1 uL/h over 7 days	0.50	10.00 Caroni and Grandes, 1990
Nerve	100 ug/mL IGF-1 concentration released over 3–4 days	Unspecified	100.00 Ehmsen and Hoke, 2020

Studies using mini-pump delivery of IGF-1 tested a variety of initial concentrations (mean = 143 μg/mL, median = 100 μg/mL, and range: 50 μg/mL – 100 mg/mL), pump rates (mean = 0.425 μL/h, median = 0.25 μL/h, and range: 0.25 – 1.05 μL/h), and release durations (mean = 26 days, median = 7 days, and range: 3 days–12 weeks). The highest dose was reported by Fansa et al. (2002) using a starting concentration of IGF-1 of 100 mg/mL dosed at a continuous pump rate of 0.25 uL/h over 28 days, a value several orders of magnitude higher than any of the other mini pump studies included in Table 5. This concentration discrepancy relative to other mini-pump studies is possibly attributable to the design of this particular study, which set out to investigate the benefits of IGF-1 on a tissue-engineered nerve graft model containing cultured, viable SCs. When the study by Fansa et al. (2002) is excluded, the reported initial optimal concentration for mini pump studies centers on a much more focused range of 0.1–100 μg/mL with a mean of 60 μg/mL and median of 75 μg/mL. Overall, all mini-pump studies included in Table 5 found a positive impact of IGF-1 on nerve regeneration.

### Local Delivery of Exogenous IGF-1 Using Hydrogels

The use of hydrogels encapsulated with varying concentrations of IGF-1 allows for a prolonged and potentially tunable release *in vivo* (Yuan et al., 2000; Mathonnet et al., 2001; Kikkawa et al., 2014; Bayrak et al., 2017). The specific hydrogel formulations that have been evaluated vary with regards to IGF-1 release kinetics, degradation rate, and biocompatibility. Despite differences in study design, the majority of hydrogel studies included in Table 6 used a water-soluble polymer oligo(poly(ethylene glycol) fumarate) (OPF) hydrogel with encapsulated gelatin microparticles (Yuan et al., 2000; Holland et al., 2005; Kikkawa et al., 2014; Bayrak et al., 2017). The extent of crosslinking within the OPF hydrogel as well as the use of encapsulated gelatin particles with variable isoelectric points allows for tunability of IGF-1 release. The cumulative release of IGF-1 by this hydrogel formulation was reported to be 95.2% ± 2.9% by Day 28, with some studies achieving a similar cumulative release within 48 h (Yuan et al., 2000; Kikkawa et al., 2014).

**TABLE 6:** *In vivo* studies using IGF-1-eluting hydrogels for local administration (BDNF, brain-derived neurotrophic factor; CNTF, ciliary neurotrophic factor; GDNF, glial cell line-derived neurotrophic factor).

**Cell Target**	**Quantity IGF-1 Used and Application Regimen**	**IGF-1 Protocol**	**Study Results (Citation)**
Nerve	0.05 ug/uL hydrogel	Hydrogels soaked in 0.05 mg/mL IGF-1 for 12 h, released over 48 h	Hydrogel-coated electrodes absorbed significantly more IGF-1 and released it over 48 h Emel et al., 2011
Nerve	40 ug/uL hydrogel	0.025–0.25 ug/uL IGF-1 (1278 ng/mL @ peak release)	Hydrogels are tunable Stitt et al., 2004
Nerve/Spinal Cord	1 ug/uL soaked gel	1 ug/uL soaked gel foam, soaked with either IGF-1, BDNF, CNTF, or GDNF	CNTF and IGF-1 soaked gels failed to prevent motoneuron death Day et al., 2001
Nerve/SC	100 ug soaked gel	100 ug IGF-1 soaked hydrogel added at time of surgical transection	Axonal order/myelination preserved in IGF-1 group, SC proliferation close to normal Day et al., 2002
Nerve	200 ug soaked plug	Two plugs soaked in 200 ug IGF-1 placed at axotomy site, spaced 12 h apart	IGF-1 induced survival in axotomized chick olfactory neurons Mohammadi et al., 2013

The hydrogels were soaked in IGF-1 solutions, with concentrations ranging from 0.05 to 1 mg/ml. The duration of soaking time and biomaterials used for fabrication differed between studies, thereby complicating further direct comparisons beyond individual consideration. Regardless of concentration of IGF-1 soaking solution, duration of soaking time, or hydrogel composition, the fundamental property in predicting utility for nerve regeneration is the sustained concentration of released IGF-1 that is reaching the site of PNI. Unfortunately, only two of the studies included in Table 6 quantified IGF-1 release *in vivo* using either fluid sampling with ELISA or radiolabeled IGF-1 (Yuan et al., 2000; Kikkawa et al., 2014). Using ELISA, one study reported significantly greater *in vivo* IGF-1 concentration, peaking at 1.25 μg/mL at Post-operative Day 1 (POD 1) and returning to the physiologic levels of the control group by POD 7 (Kikkawa et al., 2014). Using radiolabeling, the other *in vivo* quantification study reported a biphasic IGF-1 release profile with an initial burst of approximately 80% of the starting concentration of IGF-1 at 1 h followed by sustained release of the remaining 15% ± 2.9% over the subsequent 48-h period (Yuan et al., 2000). Conversely, a different study reported failure of IGF-1 to prevent motoneuron death, a finding which was noted to be contrary to previous results and required additional investigation. This study described the use of a soaked gel foam plug but did not specify the IGF-1 release profile of this material (Bayrak et al., 2017). As such, further analysis and testing is needed to determine the optimal fabrication parameters, loading strategy, and concentration of released IGF-1 required for successful local delivery via hydrogel.

## Discussion

Although numerous studies have demonstrated the benefit of IGF-1 to SCs, myocytes, and neurons *in vitro* and following PNI in animal models, several factors must be examined prior to proposing a treatment modality that is suitable for clinical translation. Besides efficacy, additional considerations include ease of regulatory clearance and safety. With regard to regulatory clearance, GH, Growth Hormone Releasing Hormone, and IGF-1 are already clinically available, FDA-approved drugs approved for other indications. With regards to safety, hypoglycemia is the most commonly seen short-term effect of IGF-1 use, although accumulation of body fat, coarsening of facial features, and lymphoid hyperplasia necessitating surgical correction have also been observed with long-term use (Contreras et al., 1995; Tuffaha et al., 2016b). Clinical trials investigating a link between malignancy and exogenous GH therapy have been equivocal, with multiple studies in children undergoing GH therapy demonstrating a low risk of associated malignancy. Additionally, GH therapy in adults has not been found to increase the risk of cancer (Yang et al., 2004; Xu et al., 2005; Chung et al., 2008; Renehan and Brennan, 2008; Svensson and Bengtsson, 2009; Tuffaha et al., 2016b). Given the potential systemic effects of IGF-1, a practical delivery system that can provide sustained release of bioactive IGF-1 to nerve and muscle tissue affected by PNI is of great importance. It will also be important to determine the minimum dose and duration required to achieve therapeutic efficacy.

Optimal dosage of IGF-1 is dependent upon its administration method. As demonstrated by Tables 1–6, there is great variation in IGF-1 dosing and frequency of administration between the various methods of delivery, with narrower ranges for ideal dosage that emerge within groups. These reported dosage ranges may serve as a useful reference point when developing and testing IGF-1 delivery strategies in pre-clinical models. Achieving the required pharmacokinetic profile for IGF-1 delivery is challenging due to the small size and short half-life of IGF-1. Therefore, designing drug delivery systems that provide targeted or local treatment of affected muscle and nerve tissue will facilitate clinical translatability of IGF-1 therapy. Local delivery of IGF-1 would reduce the side effects and potential toxicities of systemic exposure while permitting titration of loading levels to improve efficacy. However, the use of daily or frequent injections to an injury site, as described in previous studies, increases the risk of iatrogenic damage to the recovering nerve and surrounding vasculature (Caroni and Grandes, 1990; Day et al., 2001, 2002; Stitt et al., 2004; Emel et al., 2011; Mohammadi et al., 2013; Kostereva et al., 2016). In addition, the potential scarring induced by repeated local injections could preclude regenerating axons from reaching their distal targets, leading to decreased NMJ reinnervation as well as potential neuroma formation. Furthermore, the local injection of free IGF-1 without a biocompatible carrier misses an opportunity to improve its bioavailability. While the mini-pump technique provides a level of automated control over IGF-1 administration unmatched by the other previously described methods, the subcutaneous implantation of a mini-pump in a human patient introduces the risks of infection and device migration. More importantly, given the duration of time needed for regeneration to occur, the implanted pump would also likely induce a high degree of foreign body reaction resulting in fibrotic encapsulation and potential deleterious effects on the injured nerve being treated.

Despite the well-documented positive effects of IGF-1 in the setting of PNI, the major obstacle for clinical translation remains the lack of a practical delivery system that offers tunable and sustained release of bioactive IGF-1 targeted to nerve and muscle tissue downstream of the nerve injury. Such a delivery system would avoid the potential risks and side effects associated with systemic IGF-1 administration and provide a practical means of applying this treatment for both patients and clinicians (Contreras et al., 1995). The ideal IGF-1 delivery system should also demonstrate biocompatibility without inducing inflammation or encapsulation over time. In addition to the pre-soaked IGF-1 eluting hydrogels detailed in Table 6, several bioengineering approaches to local IGF-1 delivery have recently been reported in animal models. Notable amongst these studies are a delivery system which makes use of biodegradable poly(lactic-co-glycolic acid) (PLGA)/graphene oxide (GO) nanofibers embedded with immobilized IGF-1 for spinal cord repair, as well as a system of IGF-1 loaded polymeric PLGA microspheres for use in bilateral cavernous nerve injury (Santos et al., 2016; Haney et al., 2019; Pan et al., 2019).

Several bioengineered carriers have been developed in recent years for local delivery of IGF-1. Comprised of amine-terminated polyamidoamine dendrimers functionalized with polyethylene glycol (PEG), alginate, poly(γ-glutamic acid)/β-tricalcium phosphate, chitosan, gelatin, and PLGA/hyaluronic acid, these carriers have been shown to provide sustained, *in vivo* IGF-1 release profiles up to 30 days for applications including bone and cartilage regeneration (Geiger et al., 2018; Zhang et al., 2020).

The combination of nanoparticle carriers with hydrogels as a hybrid delivery system has recently come into favor for purposes including passively controlled drug release, stimuli-responsive drug delivery, site-specific drug delivery, and detoxification. The addition of a hydrogel to a nanoparticle delivery system allows for an added level of tunability as well as increased assurance that the nanoparticles remain at the local site of delivery *in vivo* (Gao et al., 2016; Norouzi et al., 2016). A promising approach being pursued by our group for repair of PNI involves encapsulation of IGF-1 into nanoparticles that provide sustained release of IGF-1 for over 6 weeks. The nanoparticles are then suspended within a biomimetic nanofiber hydrogel composite carrier to facilitate *in vivo* application and preliminary results have been encouraging (Santos et al., 2016). The approach involves injection of the composite hydrogel into the denervated target muscle and around the nerve distal to the site of injury, such that the released bioactive IGF-1 diffuses through the target tissues. Our unpublished data suggests that IGF-1 does not act on regenerating axons in gradient-dependent fashion, as uniform delivery along the distal nerve results in a robust treatment effect. However, the question of gradient dependence has not been specifically addressed to our knowledge and warrants further investigation. To achieve maximal treatment effect, IGF-1 will likely need to be delivered for the duration of the regenerative period, which can last many months or even years. It is unlikely that an engineered drug delivery system will be developed that can achieve this duration of release with a single dose. We therefore anticipate that interval ultrasound-guided reinjections will be needed, with the dosing schedule being dependent on the duration of drug release.

## Conclusion

There is strong evidence behind the supportive role of IGF-1 in recovery after PNI in animal models. IGF-1 can prevent SC apoptosis, foster axonal growth, and decrease the rate of denervation-induced muscle atrophy. Beyond the mechanistic studies that have demonstrated these positive effects *in vitro*, a number of *in vivo* studies have demonstrated efficacy by direct delivery or upregulation of IGF-1, either systemically or locally. An optimized delivery system is critically needed that can offer sustained delivery of bioactive IGF-1 to target tissues in a safe and clinically practical fashion. The optimal dosing ranges of IGF-1 vary substantially depending upon mechanism of delivery, and further work will be needed to define the dose-response relationship for any delivery method prior to clinical application.

## Author Contributions

BS, KS, NG, PH, and ST contributed to conception and design of the study. BS and ST performed the literature review, organized the database in collaboration with KS, NG, and PH, and wrote the first draft of the manuscript. BS performed preliminary stoichiometric calculations. KS, NG, and PH contributed the revisions to the manuscript draft. CQ verified all stoichiometric values and calculations and provided revisions. H-QM and AH contributed invaluable perspectives, insights, and revisions to the manuscript. ST supervised all aspects and stages of the project as corresponding author. All authors contributed to the article and approved the submitted version.

## Conflict of Interest

The authors declare that the research was conducted in the absence of any commercial or financial relationships that could be construed as a potential conflict of interest.
